# Characterization of indoor air quality in logistics warehouses in France: environmental measurements and worker exposure assessments

**DOI:** 10.1093/annweh/wxag035

**Published:** 2026-05-19

**Authors:** Laurence Robert, Romain Guichard, Jennifer Klingler

**Affiliations:** Department of Process Engineering, INRS—French National Institute for Research and Safety, 1, rue du Morvan, 54519 Vandœuvre-lès-Nancy, France; Department of Process Engineering, INRS—French National Institute for Research and Safety, 1, rue du Morvan, 54519 Vandœuvre-lès-Nancy, France; Department of Process Engineering, INRS—French National Institute for Research and Safety, 1, rue du Morvan, 54519 Vandœuvre-lès-Nancy, France

**Keywords:** indoor air quality, VOCs, aldehydes, particles, logistics warehouse, tires, employees

## Abstract

The objective of this study was to provide data on the concentrations of volatile organic compounds (VOCs), aldehydes, and total dust levels in logistics warehouses, in order to identify substances of concern for indoor air quality (IAQ) and worker exposure in these buildings. Seven warehouses were selected based on the diversity of stored products. Active sampling of VOCs, aldehydes and particles was carried out over the course of 1 d, at 3 to 5 locations within each building, as well as outdoors. Among the 7 warehouses investigated, tire logistics centers were found to have the poorest IAQ, as reflected by significantly higher concentrations of total VOCs compared with the other warehouses, ranging from 1,079 to 3,747 µg/m^3^ toluene equivalent, compared with 166 to 470 µg/m^3^ in the other sites. Based on the total VOC concentration, more in-depth investigations were conducted in the working environment involving the most degraded IAQ, namely 1 of the 2 tire warehouses. These additional analyses, including emission cell degassing of a tire sample, a detailed air characterization, and personal exposure measurements, revealed the presence of several CMR substances in these working environments, such as MIBK and aniline.

What's Important About This Paper? Occupational exposure of French workers in logistics warehouses to chemical substances is poorly documented. This study provides a dataset on the nature and concentrations of various compounds (VOCs, aldehydes, and total dust) detected in this type of work environment. The study highlights the heterogeneity of indoor air quality, with air quality depending on the type of stored products and being significantly poorer in tire storage areas compared with other environments where stored products are packaged and wrapped. These results represent a first step towards assessing worker exposure in such environments.

## Introduction

In 2023, more than 3.4 million full-time equivalent workers were employed in the retail and logistics sector in France (INSEE, 2025). For employees working in the nonfood sectors, exposure to chemical risks can often go unnoticed due to a lack of awareness. Since these workers do not handle chemicals directly but rather everyday consumer goods, few employees, employers, or even occupational health professionals may consider the potential for chemical exposure in these settings. However, volatile organic compounds (VOCs) emitted by stored consumer products contribute to the deterioration of indoor air quality (IAQ) in workplaces. Loh et al. (2006) and Nirlo et al. (2014) demonstrated the impact of products sold on the levels of VOC concentrations measured in the work environment of retail employees.

Indeed, while several studies have specifically investigated IAQ in retail stores (Loh et al. 2006; Eklund et al. 2008; Nirlo et al. 2014; Zaatari et al. 2014; Chan et al. 2015; Shang et al. 2016; Robert et al. 2021a, 2021b; Baptista et al. 2022), very few have focused on logistics warehouses. After conducting measurements in 10 retail stores throughout France, Robert et al. (2021b) revealed the presence of a large variety of VOCs in the indoor air of these stores, including carcinogenic, mutagenic, and reprotoxic (CMR) substances, with concentrations that varied significantly depending on the types of products being sold. Toluene and formaldehyde were ubiquitous, reaching concentrations of up to 252 and 53 µg/m^3^, respectively. An analysis comparing IAQ between sales areas and storage zones in 5 of the 10 stores studied (Robert et al. 2021a) revealed that air quality in storage areas was significantly poorer than in sales areas. For example, in the textile store examined in this study, total VOCs concentrations were found to be nearly twelve times higher in the storage areas compared with the sales zones. Measurements taken in the sales area of the textile store were supported by Furst et al. (2025) from a clothing shop in Portugal and by studies in underground retail spaces in South Korea (Kim et al. 2024), but these studies did not examine the storage zones. In the sports equipment store studied by Robert et al. (2021a, 2021b), toluene concentrations were over 60 times higher in the storage area compared with the sales area. IAQ in logistics warehouses is poorly documented, despite these workplaces being large storage areas with a constant influx of new products. Moreover, several characteristics of storage areas may contribute to poor air quality: overcrowding with new products arriving daily in containers, poor or nonexistent ventilation, and uncontrolled indoor temperatures. To reduce energy consumption, storage zones are typically not heated or ventilated. Although temperatures that are too low or too high affect thermal comfort, elevated temperatures further promote the off-gassing of VOCs from new goods. Indeed, environmental factors such as temperature, humidity, and ventilation characteristics significantly influence emissions (Haghighat and De Bellis 1998; Kelly et al. 1999; Ho et al. 2011; Caron et al. 2020). Recent advances in warehouse design include numerical simulations aimed at controlling temperature increases to promote better air quality (Terziev and Ivanov 2024).

These observations indicate that IAQ degradation is possible in storage spaces, highlighting the importance of this issue for the prevention of workers’ chemical exposure. Also in this context, to better understand the exposure of employees working in logistics warehouses and to provide appropriate preventive solutions, the IAQ was characterized in logistics warehouses, and additional measurements on employees were conducted.

## Materials and methods

### Description of the warehouses

Seven logistics warehouses were selected based on the type of goods stored, in order to capture the widest possible diversity. Each of the selected warehouses is located in France. The authors relied on the French occupational prevention network to identify and select the warehouses included in the study, with particular care taken to ensure diversity in both the types of products stored and the storage conditions (*ie* packaged versus unpackaged products). Seasonality was not used as a primary selection criterion for these measurement campaigns. Two warehouses store tires, 2 are dedicated to furniture and home decoration products, 1 store large building equipment such as doors, staircases, windows, or bathtubs, and another is used by a major DIY retail chain. The final warehouse stores a wide variety of miscellaneous items for the home, garden, indoor and outdoor decoration, toys, etc., serving as a distribution hub for an online retail platform. In 5 of the 7 warehouses, items are stored on metal structures with wooden pallets, while in the 2 tire warehouses, the tires are placed on fully metallic racks. In the same way, the products are generally packaged in cardboard boxes or plastic film, except for the tires and building equipment. Finally, 2 warehouses were monitored in winter and 5 in summer. However, the influence of seasonal effects has been investigated in a previous study conducted in comparable occupational environments like storage areas of retail stores selling sporting goods (Robert et al. 2021a). In this study, seasonal variations were shown to influence indoor temperature, which in turn affected the emission of chemical compounds from stored products. Specifically, increases in concentration by factors ranging from 4.0 to 5.2 for formaldehyde and from 3.6 to 6.2 for total VOCs were observed between winter and summer, corresponding to indoor temperature increases of from 11.8 to 14.2 °C.

All warehouses are built similarly, featuring a lightweight metal structure combined with fiberglass-type insulation. The surface area of each site varies according to the number of storage units in use, with each unit covering approximately 6,000 m^2^ and featuring a ceiling height of about 12 m. During the winter season, none of the warehouses are heated, and only 1 facility, which stores building equipment, is fitted with a ventilation system. The system operates via air extraction. Each section of the warehouse includes an 8,000 m^3^/h air extractor, resulting in an air exchange rate of 0.12 air volumes per hour. Typically, these facilities are ventilated by opening the loading bay doors when containers carrying storage goods are unloaded.

Building sizes range from 18,000 to 72,000 m^2^. Table 1 presents the main characteristics of the instrumented sites.

**Table 1:** Main characteristics of the warehouses.

Characteristics	E1	E2	E3	E4	E5	E6	E7
Stored products	Tires	Furniture and decoration	Furniture and decoration	Building equipment	Various products for online sales	Tires	DIY products
French department number of warehouse location	13	13	38	41	41	26	26
Number of 6,000 m^2^ cells	3	12	4	12	4	10	10
Storage area [m^2^]	18,000	72,000	24,000	72,000	24,000	60,000	60,000
Mechanical ventilation	No	No	No	Yes	No	No	No
Month and year of investigation	12/2022	12/2022	03/2023	06/2023	09/2023	06/2024	06/2024
Number of indoor measurement points	5	5	4	5	4	4	3

### Measurement strategy

In each warehouse, a measurement campaign was conducted on a single working day during typical working hours, generally between 7 AM and 6 PM. The objective of the measurement campaigns was to characterize air quality at 5 indoor locations and 1 outdoor reference point. However, in some warehouses, it was not possible to install all planned sampling points due to access restrictions. In addition, technical issues prevented the use of certain measurements (*eg* sampling pump failure, displacement of monitoring equipment by the warehouse staff). These constraints further highlight the practical challenges associated with acquiring reliable exposure data in such occupational environments, which likely contributes to the limited amount of available data reported in the literature. This selection of measurement points nonetheless allowed us to document the spatial variability within the same structure in terms of the type and concentration of VOCs, aldehydes, and particles. For comparative purposes, 1 outdoor measurement was consistently carried out.

### Sampling and analyses

All data loggers and air sampling systems were placed in wire mesh enclosures at a height of 1.4 m above the floor. These enclosures were specifically designed to prevent workers from interfering with the measurements.

ARANET 4 data loggers from TH Instrument were used to monitor air temperature, relative humidity and carbon dioxide concentrations in real time, with data transmitted every minute. Temperature measurements were accurate to ±0.3 °C within the 0 to 50 °C range. Humidity measurement were accurate to +/− 3% within the 0 to 85% range. For carbon dioxide (CO_2_) levels measured by a nondispersive infrared cell, the accuracy was 3% of the reading for concentrations under 2,000 ppmv within the 0 to 9,999 ppmv range.

#### First-stage analysis: VOCs, aldehydes, and particulate matter

Sampling of VOCs was performed with Tenax TA adsorbent tubes at a flow rate between 50 mL/min over at least 6 h, followed by thermal desorption and gas chromatography analysis in compliance with ISO 16000-6 (2021). In the initial phase, the analysis focused exclusively on the following volatile organic compounds: toluene, *o*-xylene, *m*/*p*-xylenes, styrene, and α-pinene. The concentrations are reported in μg/m^3^ of air, with a 30% uncertainty on the measured values. For all VOCs, the quantification limit (LOQ) is set at 0.1 μg/m^3^. Additionally, the analysis provided the total VOC concentration expressed as toluene equivalent for VOCs eluted between 5 and 20 carbon atoms. The results will be presented as average concentrations per chemical compound for each warehouse, calculated across all available measurement locations, together with the associated ranges of variation. These values will be compared with identify potential occupational exposure, using a total VOC concentration threshold of 1,000 µg/m^3^ expressed as toluene equivalent. When this criterion is exceeded, a more detailed investigation is conducted in the most impacted warehouse to identify the determinants of pollution and to assess workers’ exposure according to their specific tasks.

Sampling of aldehydes was performed with DNPH cartridges, operating at a flow rate of 300 to 400 ml/min over a minimum duration of 6 h. The cartridges were then analyzed using high-performance liquid chromatography (HPLC) in accordance with ISO 16000-3 (2022). Three aldehydes were primarily targeted: formaldehyde, acetaldehyde, and hexanal. Concentrations are given in μg/m^3^ of air, with an uncertainty margin of 25% on the stated value. The limit of quantification (LOQ) is 0.4 μg/m^3^ for these 3 aldehydes.

Particles were measured using a LIGHTHOUSE optical particle counter, model Handheld 3016-IAQ. Particle measurement is distributed across 6 channels ranging from 0.3 to 25 µm and is automatically converted into mass concentration in µg/m^3^. The counting efficiency is 50% for 0.3 μm particles and reaches 100% for particles above 0.45 μm. Only total dust concentration results will be reported.

Following the initial results in tire warehouses, 3 additional analyses were conducted in these environments to better understand employee exposure: a characterization of VOCs emitted by tires after degassing a sample in an emission cell, a more detailed analysis of air compounds, and exposure measurements on employees.

#### Tire degassing protocol

Two specimens measuring 2 cm by 10 cm were taken from the same new tire purchased commercially. They were placed in an emission cell swept with an inert gas at a set temperature. Two temperatures were selected for degassing: 30 and 60 °C. 30 °C represents a temperature that can be observed indoors during periods of intense summer heat. 60 °C is an extreme temperature but 1 that may be reached inside containers left in the sun awaiting unloading in the warehouse (Singh et al. 2012). In addition, this elevated temperature facilitates the degassing of the greatest amount of VOCs and offers a more complete profile of tire emissions. For these tests, samples were taken on TENAX tubes at the emission chamber outlet to identify the compounds likely to be emitted. The concentrations are given in µg/g of tire material. These tests were conducted by the TERA Environment laboratory, accredited by COFRAC for these analyses according to the ISO 16017-1 (2000) standard.

#### Further analysis of VOCs in the E6 tire warehouse

Six more compounds were measured in this warehouse, in addition to those from the initial testing: MIBK, cyclohexanone, aniline, benzothiazole, 5-methyl-2-hexanone and ethanol. MIBK was collected on a DNPH cartridge, and the remaining compounds (excluding ethanol) were trapped on TENAX tubes. These sampling conditions for both compound groups matched those previously outlined in the section on first-stage measurements. An additional sample was collected on an activated charcoal tube over 6 h at an average flow rate of 67 mL/min to capture ethanol.

#### Employee exposure in the E6 tire warehouse

In this warehouse, 3 employees were equipped in order to assess their exposure. It was decided to equip 3 employees simultaneously, each performing different tasks. A first employee, referred to as “W1”, is responsible for unloading a container and works inside it to manually remove the tires. A second employee, referred to as “W2”, receives the tires at the exit of the container, at the end of a long conveyor belt, and stores them on a mobile rack. A third employee, referred to as “W3”, moves throughout the entire warehouse to retrieve or store tires using an electric forklift truck. All 3 workers were equipped with backpacks housing air sampling pumps. Positioned at shoulder level and directed towards the face, the sampling supports were placed near the respiratory zone. The flow rates of the pumps, the type of sampling media along with the targeted compounds, as well as the sampling duration characteristic of a work task for each of the 3 employees, are presented in Table S1.

## Results and discussion

### Indoor environmental parameters

Table S2 shows the average and the minimum/maximum variations of air temperature, as well as the average and minimum/maximum CO_2_ concentration in all 7 warehouses of the panel on the day of the measurements. To allow comparison, the matching values for outdoor air are provided. The temperatures in the first 2 warehouses, measured in winter, are markedly lower than in the others, recorded at 12.8 and 15.7 °C. For the other 5 warehouses, where measurements were carried out during spring or summer, average temperatures range between 18.5 and 24.3 °C. The average relative humidity showed limited variability across the different warehouses and measurement periods, ranging from 42.5% to 49.4%, with the exception of warehouse E5, where measurements were conducted on a stormy day with a higher outdoor relative humidity of 71%. Under these temperature and humidity conditions, no clear indication of summer thermal discomfort for warehouse workers can be inferred, although a comprehensive assessment of thermal comfort would require additional parameters, including workers’ metabolic rate, clothing insulation, air velocity, and mean radiant temperature. The absence of thermal discomfort does not apply to workers unloading shipping containers. During summer periods, container internal temperatures can reach very high values potentially up to 60 °C (Singh et al. 2012). Combined with intense radiant heat from container walls and high physical workload, these conditions may lead to situations of significant thermal stress. Regarding CO_2_ concentrations, there is a clear correlation between the corresponding indoor and outdoor values. Considering the size of these workspaces, the presence of employees does not affect indoor CO_2_ levels through their respiration. For warehouse E3, an abnormally elevated outdoor CO_2_ concentration of 560 ppmv was recorded, resulting from the placement of the external sensor near a heavily trafficked motorway on the measurement day.

### Concentrations of aldehydes, VOCs and dust measured in preliminary analysis

**Figure 1:**
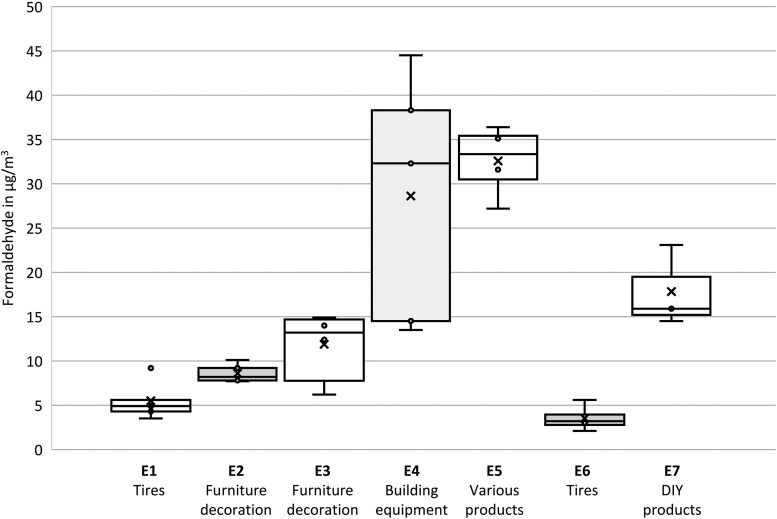
Distribution of formaldehyde concentrations per warehouse expressed in µg/m^3^.

**Figure 2:**
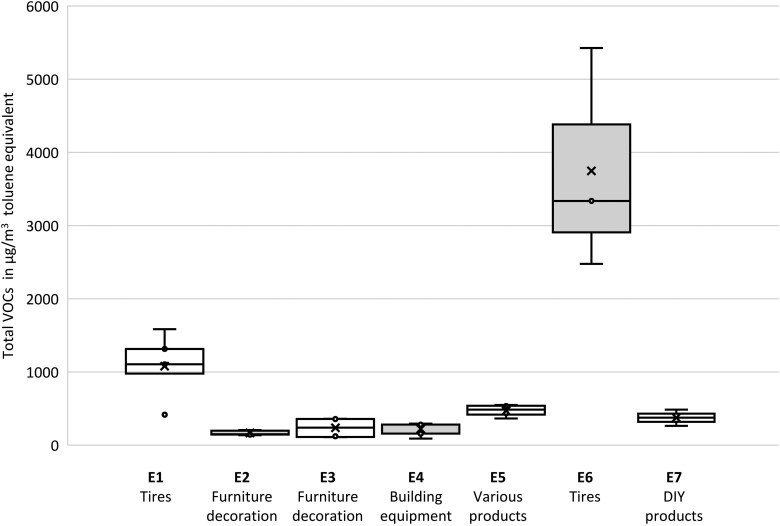
Distribution of total VOC concentrations per warehouse expressed in µg/m^3^ toluene equivalent.

Table 2 presents the average and minimum/maximum concentrations of VOCs and aldehydes. These results are displayed using boxplots for formaldehyde concentrations in Fig. 1 and for total VOC concentrations in Fig. 2. Among the 3 quantified aldehydes, formaldehyde averages range from 3.5 to 32.6 µg/m^3^, with a maximum local value of 44.5 µg/m^3^ observed in warehouse E4, which stores large construction equipment such as doors, stairs, windows, and bathtubs (Fig. 1) while the maximum mean concentration was obtained in warehouse E5. These concentrations may be explained by the larger amount of wood-based materials, varnishes, and MDF present, as well as the highest indoor temperature recorded (24.3 °C) (Caron et al. 2020; Garras et al. 2021). It is also observed that in the 2 tire storage warehouses E1 and E6, the formaldehyde concentrations are clearly the lowest, at 3.5 and 5.5 µg/m^3^, and close to the average outdoor level of 2 µg/m^3^. The average formaldehyde concentrations in this selection of warehouses, from 3.5 to 32.6 µg/m^3^, are in line with those recorded in retail stores by Robert et al. (2021a, 2021b), which ranged from 8 to 28 µg/m^3^ using the same measurement methodology. Acetaldehyde concentrations range on average from 1.8 to 20 µg/m^3^, with a maximum value of 33.2 µg/m^3^. Hexanal, for its part, varies on average between 9 and 19.5 µg/m^3^ and was not found in the 2 warehouses storing tires, E1 and E6. Finally, all the measured concentrations are below the occupational exposure limits provided by the French National Research and Safety Institute (INRS). As a reminder, for formaldehyde, which is classified as a CMR (Carcinogenic, Mutagenic, or Reprotoxic) substance, the occupational exposure limit (OEL) is 625 μg/m^3^ for an 8-h exposure (INRS, 2025). Furthermore, none of the concentrations exceeded the IAQ guideline set by the French Agency for Food, Environmental and Occupational Health & Safety (ANSES, 2024), which is 100 μg/m^3^ for short-term exposure (30 min) to formaldehyde.

**Table 2:** Indoor concentrations of aldehydes and VOCs (µg/m^3^) and total VOCs (µg/m^3^ toluene equivalent) by warehouse type (average concentration in bold; minimum and maximum in brackets).

		E1	E2	E3	E4	E5	E6	E7	Outdoor
Number of measurements		5	5	4	5	4	3	3	7
Compound	CAS No.								
Formaldehyde	50-00-0	**5.5** [3.5; 9.2]	**8.6** [7.7; 10,1]	**11.9** [6.2; 14.9]	**28.6** [13.5; 44.5]	**32.6** [27.2; 36.4]	**3.5** [2.1; 5.6]	**17.8** [14.5; 23.1]	**2** [<LQ; 4.8]
Acetaldehyde	75-07-0	**1.8** [2.1; 3.9]	**4.3** [3.5; 5.7]	**10.2** [5; 15.7]	**20.0** [5.2; 33.2]	**11.5** [10.5; 12.9]	**16.1** [11.8; 20.9]	**13.5** [13.1; 13.8]	**0.8** [<LQ; 3.9]
Hexanal	66-25-01	**<LQ**	**9.0** [6.4; 13.2]	**19.5** [7.8; 34.7]	**18.0** [6; 33.8]	**13.6** [12.3; 15.4]	**<LQ**	**13.5** [13.1; 13.8]	**<LQ**
Toluene	108-88-3	**22.6** [16.1; 29.9]	**3.7** [3.1; 4.4]	**2.6** [1.4; 3.9]	**3.6** [1.8; 5.0]	**14.9** [10.1; 18.4]	**49.0** [24.4; 83.9]	**2.5** [2.4; 2.6]	**0.6** [0.2; 1.6]
Styrene	100-42-5	**61.7** [18.4; 87.9]	**4.3** [3.1; 5.4]	**0.5** [0.1; 1.2]	**10.0** [1.6; 21.1]	**24.1** [21.5; 28.3]	**176.6** [109.4; 293.4]	**2.0** [1.9; 2.0]	**0.1** [<LQ; 0.6]
*m*-,*p*-,*o*-Xylenes	95-47-6 & 108-38-3 & 106-42-3	**44.5** [14.4; 67.2]	**6.3** [5.4; 7.6]	**1.7** [1; 2.2]	**9.8** [1; 25.5]	**36.4** [32.2; 40.5]	**161.8** [101.9; 246.9]	**2.3** [2.2; 2.3]	**1.5** [0.1; 7.2]
a-Pinene	80-56-8	**4.9** [3.1; 6.6]	**37.7** [29.4; 46.7]	**67.3** [25.3; 13.7]	**76.7** [31.6; 10.1]	**75.0** [51.4; 90.9]	**3.5** [1.4; 4.8]	**61.6** [45.2; 78.0]	**0.1** [<LQ; 0.3]
Total VOCs C5-C20	—	**1,079** [415; 1,584]	**166** [134; 206]	**236** [108; 359]	**220** [89.9; 293]	**470** [363; 549]	**3,747** [2,477; 5,427]	**374** [263; 485]	**49.7** [2.0; 254.7]

Regarding the quantified VOCs, concentrations ranged from 0.5 to 176.6 µg/m^3^ across all compounds combined. In general, a different chemical signature is observed between the warehouses storing tires (E1 & E6) and the other 5 warehouses. In the 2 tire warehouses, besides the lowest formaldehyde concentrations and the absence of hexanal, there is also a low amount of α-pinene, 4.9 and 3.5 µg/m^3^ compared with 37.7 to 76.7 µg/m^3^ in the other facilities. The use of completely metal storage racks, lacking wooden pallets, might explain the low terpene concentrations observed in the 2 tire warehouses. However, these warehouses also show significantly higher levels of styrene, 61.7 and 176.6 µg/m^3^ compared with 0.5 to 24.1 µg/m^3^ in the other warehouses, higher levels of xylenes, 44.5 and 161.8 µg/m^3^ compared with 1.7 to 36.4 µg/m^3^, as well as higher levels of toluene.

Concentrations of total VOCs, which are a good indicator of IAQ, also reveal a decline in IAQ within the tire warehouses, with values of 1,079 and 3,747 µg/m^3^ toluene equivalent compared with 166 to 470 µg/m^3^ toluene equivalents across the other warehouses. The study panel shows average total VOCs concentrations between 166 and 3,747 μg/m^3^ toluene equivalent, evidencing large variability across the 7 warehouses, comparable to Robert et al. (2021b) observations in commercial environments, which reported total VOCs concentrations ranging from 218 to 3,114 μg/m^3^ toluene equivalent. In France, no directive exists concerning IAQ standards for total VOCs.

For each environment, the 3 most abundant compounds identified in the screening—excluding those subsequently quantified—is reported in Table S3. Notably, benzene was not identified among the compounds detected in the screening analysis. In warehouses storing packaged goods and/or furniture, where wood-based materials are prevalent (*eg* wooden pallets or building equipment), terpenes were predominantly identified, including β-pinene, 3-carene, cedrene, and limonene. In contrast, in the 2 tire warehouses, the compounds specifically identified through screening were methyl-isobutyl-ketone (MIBK), cyclohexanone, benzothiazole, and aniline; these compounds were therefore selected for quantification.

Tires appear to have a greater impact on IAQ than other stored consumer goods. It is made clear that these, along with the building equipment stored in warehouse E4, are the only products that are neither carton-packed nor film-wrapped. In fact, the other stored goods come in cartons and are kept as is on storage racks, without unpacking. This packaging promotes the containment of VOCs present in consumer goods; these VOCs are more frequently released upon opening the cartons and unpacking the goods (Robert et al. 2021a).

Finally, total particle measurements were carried out and the average and extreme concentrations of total dust are presented in Table 3, revealing concentrations ranging from 20.8 to 256.3 µg/m^3^. In France, the limit value for dust without specific effect (PSES) is set at 4,000 μg/m^3^ for inhalable dust (Légifrance 2023). The detected concentrations are significantly under this regulatory limit. The monitoring instruments used also allow the determination of particle size fractions PM_1_, PM_2.5_, and PM_10_, corresponding to the proportion of particles in the total sample with aerodynamic diameters below 1, 2.5, and 10 µm, respectively. The particle size fractions are presented in Table 3. Regarding PM_10_, the profiles of the 7 warehouses are comparable, with a large proportion of particles below 10 µm, ranging from 72.1% to 87.9% of the total dust concentration. Warehouses E4 and E6 nevertheless stand out from the rest of the dataset due to a higher proportion of finer particles, with PM_1_ fractions of 45.9% and 24.1%, respectively, compared with <3.5% in the other environments. All these particulate fractions are inhalable by workers; however, particles smaller than 4 µm, thus including PM_1_ and PM_2.5_, can penetrate more deeply into the respiratory tract, reaching the pulmonary alveoli, rather than being deposited in the upper airways.

**Table 3:** Indoor average and range of total particle concentrations (µg/m^3^) depending on the warehouse type and average proportion of PM_1_, PM_2.5_ and PM_10_ (%).

Total PM concentrations in the warehouses (in µg/m^3^)	Number of measurements	Concentration range	AVERAGE	Proportion of PM_1_	Proportion of PM_2.5_	Proportion of PM_10_
E1	4	[55.6; 258]	109.1	3.1	5.6	72.1
E2	4	[20.5; 55.6]	39.2	3.5	6.6	80.6
E3	3	[39.8; 80.7]	66.6	1.4	4.2	78.1
E4	4	[26.8; 117]	55.8	45.9	48	78.7
E5	4	[38.9; 437]	187.7	1.3	3	78.2
E6	4	[14.2; 33.4]	20.8	24.1	29.0	87.9
E7	3	[176; 391]	256.3	1.0	4.4	75.7

### Exploring the tire sector: IAQ characterization and measurements of employee exposure

In view of these initial results, particularly those concerning VOCs, it was decided to carry out a more in-depth characterization of IAQ as well as the exposure of workers in a tire logistics warehouse. For this purpose, 3 measurement campaigns were carried out: first, degassing of tire specimens to determine which specific VOCs may be emitted by tires; then simultaneously, a deeper characterization of VOCs in the air of a tire warehouse and the exposure monitoring of several workers employed there. The in-depth characterization of VOCs and employee exposure was conducted in warehouse E6.

The degassing tests reveal 2 pieces of information: the identification of VOCs specifically emitted by tires, which are not necessarily quantified at first when assessing IAQ, and the impact of temperature on their emission rate. Numerous compounds were quantified during these tests: aniline, benzothiazole, MIBK, cyclohexanone, naphthalene, etc. They are listed in Table S4. Some of these compounds are classified as carcinogenic, mutagenic or reprotoxic, such as aniline and MIBK. Degassing at higher temperatures makes it possible to observe the impact of temperature on the emissions of these substances or on the appearance of others. For example, in the tests carried out at 60 °C, compared with those at 30 °C, aniline and benzothiazole increased by a factor of 25, from 0.07 to 1.75 µg/g of tire and from 0.42 to 10.73 µg/g of tire, respectively. MIBK and cyclohexanone also increased, although to a lesser extent, by a factor of around 1.5 between the tests at 30 and 60 °C.

Given these results and with the aim of better characterizing IAQ in a tire logistics warehouse, it was decided to add 4 compounds to the initial measurements: aniline, MIBK, cyclohexanone and benzothiazole. These compounds were chosen either because they are CMR, like aniline for example, or because they appeared significantly during the test conducted at 30 °C, which represents a possible working temperature, especially in containers during summer.

Table 4 reveals very significant concentrations of MIBK, ranging from 699 to 1,547 µg/m^3^, in warehouse E6, as well as of cyclohexanone, aniline, and benzothiazole, whose average concentrations are 330, 302, and 277 µg/m^3^, respectively. These tire-specific compounds were quantified in clearly higher amounts than the VOCs initially selected in this study to assess IAQ. This reveals a distinctive chemical signature specific to tires.

**Table 4:** Concentrations of formaldehyde and major VOCs of interest in the E6 tire warehouse in µg/m^3^.

Volatile organic compound	CAS number	No. of measurements—warehouse	Concentration range	Average	Outdoor
Formaldehyde	50-00-0	4	[2.1; 5.6]	3.5	1.4
MIBK	108-10-1	4	[699; 1,547]	1,126	2.3
Cyclohexanone	108-94-2	3	[195; 522]	330	< LOQ
Toluene	108-88-3	3	[24; 83.9]	49.0	0.2
Aniline	62-53-3	3	[232; 440]	302	< LOQ
Benzothiazole	95-16-9	3	[236; 345]	277	0.9
*m*-,*p*-,*o*-Xylenes	95-47-6 & 108-38-3 & 106-42-3	3	[102; 247]	162	0.1
Styrene	100-42-5	3	[109; 293]	177	< LOQ
Ethanol	64-17-5	4	[6,308; 15,475]	9,947	< LOQ
Total VOCs	—	3	[2,477; 5,427]	3,747	254

Table 5 presents the measurements collected on workers’ exposure. When comparing Tables 4 and 5, it appears that worker exposure can sometimes be higher than the levels measured in indoor air. This is particularly the case for MIBK. In the warehouse, MIBK concentrations vary between 699 and 1,547 µg/m^3^ (Table S4), whereas for workers W1 and W2, exposure levels are much higher: 4,358 and 1,759µg/m^3^ (Table 4). For worker W3, who operates throughout the entire warehouse, his exposure to MIBK (1,259 µg/m^3^) is very similar to the average concentration of the compound in the warehouse air (1,126 µg/m^3^). This observation also applies to most other compounds: formaldehyde, aniline, *m*,*p*,*o*-xylenes, cyclohexanone, and others.

**Table 5:** Concentrations of formaldehyde and VOCs (in µg/m^3^) and total VOCs (µg/m^3^ toluene equivalent) measured on 3 employees of the E6 tire warehouse.

Volatile organic compound	CAS number	Employee W1	Employee W2	Employee W3
Formaldehyde	50-00-0	10.2	11.8	3.7
MIBK	108-10-1	4,358	1,759	1,259
Cyclohexanone	108-94-2	533	306	412
Toluene	108-88-3	184	75.6	65.7
Aniline	62-53-3	538	564	441
Benzothiazole	95-16-9	253	306	362
*m*-, *p*-,*o*-Xylenes	95-47-6 & 108-38-3 & 106-42-3	528	206	228
Styrene	100-42-5	275	302	268
Benzene	71-43-2	36.2	7.3	6.7
Ethanol	64-17-5	13,655	n.m.	3,354
Total VOCs in toluene equivalents	—	8,188	7,427	5,386

Among the quantified compounds, measurements show that workers are exposed to a mixture of carcinogenic substances: formaldehyde, aniline, MIBK, and benzene. For an 8-h workday, the occupational exposure limits are 625 μg/m^3^ for formaldehyde (INRS, 2025), 7,740 for aniline (INRS, 2022), 83,000 for MIBK (INRS, 2023), and 1,650 for benzene (INRS, 2024a). Even if the measured averages are under these limit values, the mixture of compounds could still create risks for employees. The MiXie France tool (INRS, 2024b) was used to assess the concentrations measured on worker W1. The tool cannot determine the carcinogenic risk, as the additivity hypothesis for this type of risk is not valid, but it highlights the carcinogenic potential of the 4 compounds; it also alerts to the sensitizing effects of 2 substances (aniline and formaldehyde), the presence of an endocrine disruptor, and estimates a 17% risk of ocular damage for workers exposed to this atmosphere.

During tire manufacturing, several processing steps may involve the use of alcoholic solvents, such as mould-release agents, cleaning solvents for moulds or surfaces, or formulations of waxes, resins, or surface-applied additives. Traces of ethanol may therefore remain trapped within the rubber matrix or adsorbed on the surface and subsequently be progressively desorbed after manufacturing, particularly in poorly ventilated spaces. This mechanism could plausibly explain the ethanol concentrations measured in the present study (Tables 4 and 5).

A second conclusion from these exposure measurements concerns the impact of specific work tasks on the exposure level of a worker. Worker W1, who is in constant contact with tires and works inside a container, an enclosed space with limited air renewal and a high tire density, is significantly more exposed than the other 2. For example, he is exposed to a total VOCs concentration of 8,188 µg/m^3^ expressed as toluene equivalent, while worker W2 is exposed to 7,427 µg/m^3^ and worker W3 to 5,386 µg/m^3^, also expressed as toluene equivalent. W2 also works with tires all day, but he's in the warehouse, which is bigger and better ventilated, resulting in less exposure. The warehouse reception zone's volume (1,500 m^2^ or one-quarter of a cell, with 11.5 m ceilings and dock doors that may remain open) allows for compound dilution, explaining W2's lower exposure compared with W1. Finally, the third worker, W3, does not manually handle tires. He loads and unloads mobile racks to transport them to storage areas and moves throughout the entire warehouse; his exposure closely matches ambient air levels.

## Conclusion

This study reports on the concentrations of VOCs, aldehydes, and particles measured in 7 French logistics warehouses. The measured formaldehyde concentrations ranged from 3.5 to 32.6 µg/m^3^ on average. The most significant initially measured VOCs were styrene, detected up to 293.4 µg/m^3^, and α-pinene at 246.9 µg/m^3^. Total particle levels showed average concentrations between 14.2 and 437 µg/m^3^. Even though these initial series of measurements reveal concentrations of VOCs, aldehydes, and total particles well below health reference thresholds, they showed that tire warehouses exhibited poorer air quality compared with other warehouses storing packaged and film-wrapped products. The average total VOCs concentration in these tire-specific warehouses ranged from 1,079 to 3,747 µg/m^3^, compared with 166 to 470 µg/m^3^ for the other 5 warehouses in the panel.

In this context, additional measurements were conducted within the tire sector. Off-gassing tests on tire samples revealed the presence of VOCs specifically emitted by this product type. Aniline, MIBK, cyclohexanone, and benzothiazole were identified as VOCs specific to tires and significantly emitted by them. These compounds were included in the more detailed characterization of air in a tire warehouse. Their concentrations exceeded those of the initially investigated compounds. MIBK concentrations averaged around 1,126 µg/m^3^, while aniline, benzothiazole, and cyclohexanone were quantified at approximately 302, 277, and 330 µg/m^3^ respectively.

Finally, measurements on 3 employees performing different tasks in the E6 tire warehouse completed this study. It appears that these workers are generally exposed to concentrations higher than those measured in the warehouse air. Exposure to a mixture of compounds, including carcinogenic, mutagenic, and reprotoxic (CMR) substances, was identified, despite all individual concentrations being below the French occupational exposure limits. Moreover, the specific job tasks performed by these employees have a substantial impact on their exposure. Also, worker W1, who works exclusively inside the container, is clearly more exposed than the worker who operates throughout the entire warehouse performing picking or storage tasks. In addition to the observation of higher chemical exposure, the manual work of the tire unpacker carried out in a confined space can lead to other occupational risks, such as heat stress during the summer months. This multiple exposure deserves to be further investigated; however, it was not addressed in the present study that focused on chemical exposure, which constitutes a first limitation regarding the assessment of workers’ exposure in logistics warehouses.

In addition, this preliminary study was limited to only 7 warehouses. Although the results provide an initial overview, other exposure situations or IAQ levels may occur in different logistics warehouses and reveal additional issues. A larger panel of warehouses, including a wider variety of stored products, would therefore be useful to complement these first observations. For instance, workers’ exposure during the storage of bulk cereals (Rodriguez-Zamora et al. 2017; Cui et al. 2021) or large sports equipment such as surfboards or paddleboards, as well as resin-based products such as bathtubs or shower trays associated with styrene emissions (Carlo et al. 2007), would merit further investigation.

Another limitation of this work is that measurements were conducted during a single season and over 1 single day per warehouse. These preliminary results would benefit from complementary measurements carried out at different times in order to assess temporal variability in IAQ. Furthermore, a maximum of 5 indoor measurement locations per warehouse were investigated, whereas in large-volume spaces, concentrations may vary significantly within the same warehouse depending, for example, on proximity to loading dock doors. Nevertheless, these observations provide useful insights into the methodology to be adopted for future measurement campaigns.

## Supplementary Material

wxag035_Supplementary_Data

## Data Availability

The data underlying this article are available in the article and in its online Supplementary material.
