# Supplementary material for: Characterization of indoor air quality in logistics warehouses in France: environmental measurements and worker exposure assessments
Source: Ann Work Expo Health. 2026 May 19;70(4):wxag035. doi: 10.1093/annweh/wxag035 (PMC13186187; doi:10.1093/annweh/wxag035)
Supplement: wxag035_Supplementary_Data [file wxag035_supplementary_data.pdf]

Characterization of Indoor Air Quality in Logistics Warehouses in France: Environmental Measurements and Worker Exposure Assessments

Laurence Robert*, Romain Guichard and Jennifer Klingler

Department of Process Engineering, INRS – French National Institute for Research and Safety, Vandœuvre-lès-Nancy, France

* Corresponding author: Dr Laurence Robert, laurence.robert@inrs.fr

Supplementary material

Table S1: Sampling details on employees including duration (min) and flow rate (ml/min)

	Type of support	Target compounds	Employee W1 (ml/min)	Employee W2 (ml/min)	Employee W3 (ml/min)
Sampling duration			64	63	357
Sampling rate	TENAX tube	Other VOCs	134	68	56
Sampling rate	DNPH cartridge	Aldehydes	398	402	429
Sampling rate	Activated charcoal tube	Ethanol	170.5	196.5	215.5
Sampling rate	CARBO Graph 4 tube	Benzene	63.5	53.5	68.5

Table S2: Temperature in °C and concentration of CO_2 in ppmv in indoor and outdoor air (building average in bold; minimum and maximum depending on measurement location in brackets)

	E1	E2	E3	E4	E5	E6	E7
Number of measurements	4	5	4	5	3	4	3
Indoor air temperature (°C)	**15,7**<br>[14.9 ; 16.6]	**12.8**<br>[11.8 ; 14.5]	**18.5**<br>[17.4 ; 21.6]	**24.3**<br>[23.7 ; 25]	**23.5**<br>[23.2 ; 23.7]	**23.7**<br>[23.6 ; 23.9]	**22.1**<br>[21.8 ; 22.4]
Outdoor air temperature (°C)	**15.6**	**6.9**	**22.4**	**26.1**	**22.7**	**24.2**	**24.2**
Indoor air humidity (%)	**44.8**<br>[36.4 ; 45.6]	**49.4**<br>[46.3 ; 52.2]	**42.5**<br>[38.7 ; 44.7]	**44.8**<br>[42.4 ; 47.1]	**59.7**<br>[58.5 ; 60.8]	**44.6**<br>[43.1 ; 45.9]	**44**<br>[43.7 ; 44.3]
Outdoor air humidity (%)	**48.1**	**64.2**	**35.9**	**43.4**	**71.9**	**43.4**	**43.4**
Indoor CO_2 concentration (ppmv)	**466**<br>[452 ; 480]	**469**<br>[458 ; 487]	**441**<br>[416 ; 466]	**431**<br>[424 ; 437]	**476**<br>[462 ; 494]	**472**<br>[419 ; 606]	**462**<br>[416 ; 535]
Outdoor CO_2 concentration (ppmv)	**410**	**409**	**560**	**385**	**n.m.**	**452**	**452**

n.m.: not measured

Table S3: Three main compounds present in the screening, expressed as a percentage of the total VOC concentration

	Compounds	CAS number	Number of measurments	Average percentage of the total VOC concentration	Percentage range
E1	5 methyl, 2 hexanone	110-12-3	5	29.1 %	[28.3 ; 30.3] %
	MIBK	108-10-1	5	13.2 %	[12.3 ; 13.8] %
	Benzothiazole	95-16-9	5	12.1 %	[10.2 ; 14.8] %
E2	3-carene	13466-78-9	5	10.0 %	[9.1 ; 10.5] %
	Cedrene	11028-42-5	5	6.3 %	[3.3 ; 9.1] %
	β-pinene	127-91-3	5	3.6 %	[2.7 ; 4] %
E3	3-carene	13466-78-9	4	21.3 %	[15.4 ; 27.2] %
	Limonene	138-86-3	4	2.7 %	[1.7 ; 3.5] %
	β-pinene	127-91-3	4	2.4 %	[2.5 ; 2.9] %
E4	3-carene	13466-78-9	4	23.4 %	[23.0 ; 23.9] %
	β-pinene	127-91-3	5	6.0 %	[4.5 ; 8.9] %
	Benzaldehyde	100-52-7	5	2.3 %	[1.3 ; 2.9] %
E5	Cedrene	11028-42-5	4	11.7 %	[10.3 ; 13.2] %
	3-carene	13466-78-9	4	4.5 %	[4.2 ; 5.1] %
	(+)-b-funebrene	79120-98-2	4	3.4 %	[2.8 ; 4.1] %
E6	Cyclohexanone	108-94-1	3	8.6 %	[7.9 ; 9.6] %
	Aniline	62-53-3	3	8.2 %	[7.0 ; 9.4] %
	Benzothiazole	95-16-9	3	7.8 %	[6.3 ; 9.5] %
E7	β-pinene	127-91-3	2	7.5 %	[3.9 ; 11.1] %
	Acetone	67-64-1	2	2.8 %	[1.9 ; 3.7] %
	Nonanal	124-19-6	2	2.2 %	[1.9 ; 2.6] %

Table S4: Main VOCs emitted by a tire in µg/g of tire

**Volatile Organic Compound (VOC)**	CAS Number	**Degassing at 30 °C**	**Degassing at 60 °C**
Aniline	62-53-3	0.07	1.75
Benzothiazole	95-16-9	0.42	10.73
MIBK	108-10-1	0.34	0.48
Ethyl Octanoate	106-32-1	0.05	1.15
Cyclohexanone	108-94-2	0.33	0.53
4-(1,1-Dimethylethyl)cyclohexanone	98-53-3	< LQ	0.74
Naphthalene	91-20-3	< LQ	0.35
3-heptene,2,2,4,6,6-pentamethyl	123-48-8	< LQ	0.37
Benzenamine, N-(2,2-dimethylpropyl)-N-methyl	53927-61-0	< LQ	1.06
1,2-Dihydro-2,2,4-trimethylquinoline	147-47-7	< LQ	4.42
4-(1,1,3,3-Tetramethylbutyl)phenol	140-66-9	< LQ	6
Diphenylamine	122-39-4	< LQ	1.47
**Total VOCs**	---	**1.51**	**37.58**
